# A creative approach to the diagnosis and treatment of low back pain: case report and review of the literature

**DOI:** 10.1093/jscr/rjaf403

**Published:** 2025-06-09

**Authors:** Megan M Finneran, Angeli Mittal, Amit Vyas, Jason Seibly

**Affiliations:** Department of Neurosurgery, Carle BroMenn Medical Center, 1304 Franklin Ave, Normal, IL 61761, United States; Stritch School of Medicine, 2160 S 1st Ave, Maywood, IL 60153, United States; Department of Radiology, Carle BroMenn Medical Center, 1304 Franklin Ave, Normal, IL 61761, United States; Department of Neurosurgery, Carle BroMenn Medical Center, 1304 Franklin Ave, Normal, IL 61761, United States

**Keywords:** SPECT-CT, osteoblastic, lumbosacral fusion, low back pain

## Abstract

Chronic low back pain is a common complaint seen in the neurosurgical clinic that is often difficult to manage. A relatively new addition to the diagnostic armamentarium is single photon emission computed tomography-computed tomography (SPECT-CT). We present the case of a 76-year-old male who presented with long-standing right-sided low back pain that had progressed to the point of making him wheelchair-bound. SPECT-CT demonstrated increased uptake within an osteoblastic-appearing focus between the right L5 transverse process, sacral ala, and anterior sacroiliac joint. The patient underwent right SI joint fusion with pedicle screw fixation at L4 and L5, connected to a right-sided iliac screw. The patient had significant resolution of his pain and was able to ambulate postoperatively for the first time in several years. This creative treatment approach aimed to specifically target the problematic lesion and successfully provided clinical relief.

## Introduction

Low back pain is a complaint that plagues patients and their treating physicians. The cause of pain is often difficult to clearly elucidate and may encompass a combination of facet joints, discs, nerve impingement, ligaments, muscles, and spinal instability [1]. Accurate identification of the pain-generating source is a crucial part of formulating a treatment plan. Diagnostic modalities may include computed tomography (CT), magnetic resonance imaging, and diagnostic injections. Single photon emission computed tomography-computed tomography (SPECT-CT) has shown to be a relatively new, effective diagnostic modality [2].

When SPECT-CT can identify a source, the treatment plan should cater to addressing the specific pathology. We present a case of an osteoblastic lesion within the lumbosacral spine that was treated with combination sacroiliac fusion and pedicle screw fixation.

## Case report

A 76-year-old male with past medical history of diabetes, hypertension, hyperlipidemia, history of smoking (quit 33 years prior), bladder cancer controlled without chemotherapy, and atrial fibrillation presented to the neurosurgical clinic with severe right-sided low back pain. The pain was focal over the right sacroiliac (SI) joint. He was wheelchair-bound due to the severity of the pain. He denied any pain on the left side and denied a radicular component to his pain. An SI joint steroid injection previously performed had provided zero relief.

On examination, he had no focal neurological deficits. The strength in bilateral lower extremities was 5/5 in all muscle groups. Sensation was intact. He had severe tenderness to palpation over the right side of the low back over the SI joint and posterior superior iliac spine (PSIS). Straight leg raise on the right elicited pain in the back.

### Imaging findings

**Figure 1:**
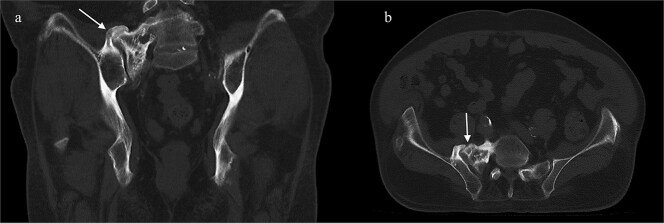
Coronal CT pelvis (left) and axial (right) demonstrated hyperostosis along the right SI joint suggestive of an osteoblastic lesion between the L5 transverse process and sacral ala (arrows).

**Figure 2:**
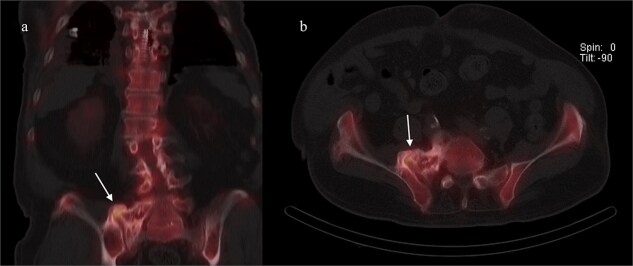
Coronal (left) and axial (right) views of pelvic CT-SPECT highlighted increased uptake along the right SI joint, suggestive of inflammation (arrows).

A CT of the pelvis showed significant hyperostosis along the anterior right SI joint and a pseudoarthrosis between the L5 transverse process and the sacral ala that was osteoblastic-appearing in nature (Fig. 1). A SPECT-CT demonstrated increased uptake along the same area, suggestive of inflammatory degenerative changes (Fig. 2).

### Procedure

The patient was placed in the prone position with horizontal gel rolls. Intraoperative neuromonitoring was used to monitor lower extremity somatosensory evoked potentials and electromyography.

A right-sided paramedian incision was made, just medial to his previously placed spinal cord stimulator. Bovie cautery was used to incise the subcutaneous and lumbar fascia. Blunt finger dissection was used to spread the muscle exposing L4-5 and L5-S1 facet complexes. A percutaneous neuronavigation frame was placed into the left PSIS. O-arm navigation was used for navigation. Using a navigated drill, the right L4 and L5 pedicles were cannulated. A 4.5 mm tap was then placed, with 6.5 mm diameter, 45 mm length screws placed in the right L4 and L5 pedicles.

The original plan was to place an S2-trans-iliac screw, but the trajectory was not well aligned. Instead, an iliac screw was placed. The medial aspect of the PSIS was cannulated with a navigated drill. A 5.5-mm tap was used, followed by a 7.5-mm tap. A 10.5 mm diameter, 90 mm length Granite (SI-bone company) iliac screw was placed.

The navigated drill was used to drill out the osteoblastic lesion along the lateral right L5 vertebral body. A 12 mm Rialto tap was used to drill through the osteoblastic mass; the bone from the tap was sent to pathology for analysis, which later revealed no neoplastic process.

A rod was contoured to connect the three screws. Locking caps were placed and tightened to final torque.

**Figure 3:**
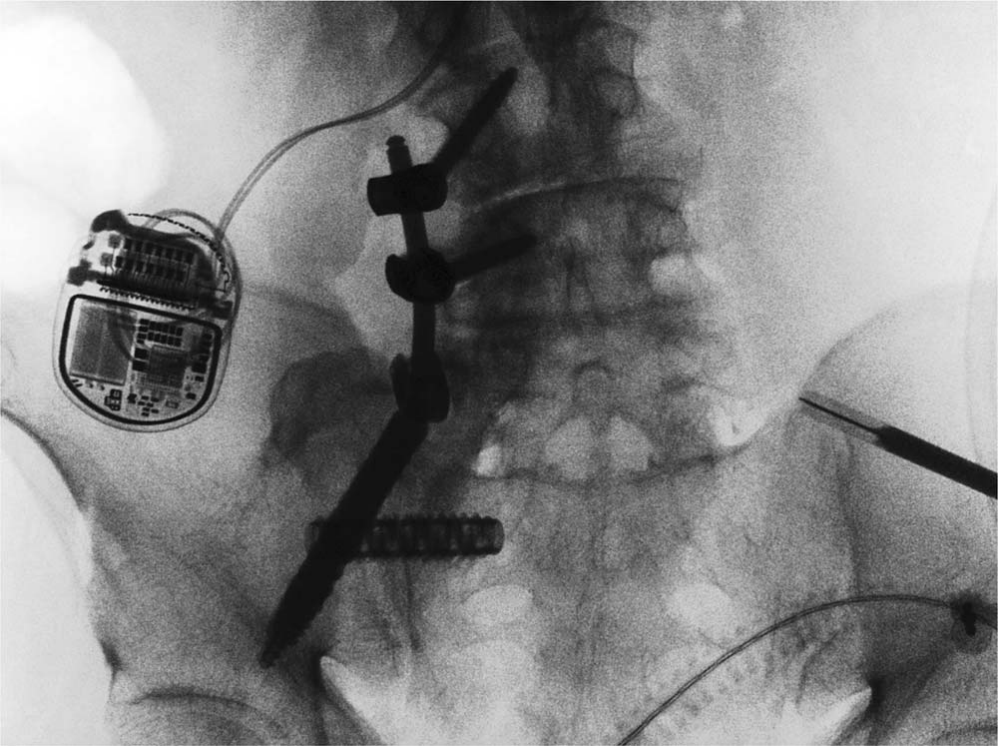
Intraoperative O-arm spin confirmed placement of the right L4 and L5 pedicle screws, as well as the right iliac screw and right sacroiliac implant. The left-sided navigation clamp can be visualized. The patient’s spinal cord stimulator (seen in the figure) was carefully avoided. One can also see the pronounced osseous abnormality along the right lower spine, extending to the sacrum and medial ilium.

Next, a minimally invasive right SI joint bolt was placed. A second, 1-in. incision was made over the right lateral iliac fossa. A navigated drill was used to cannulate a pilot hole through the right ilium, crossing the sacroiliac joint, into the right sacral ala. A 12 mm tap was then used, and a 12 mm diameter, 50 mm length Rialto SI joint fusion implant was inserted across the right sacroiliac joint. A final O-arm scan was performed to confirm good position of all implants (Fig. 3).

Both incisions were irrigated with antibiotic solution and closed in standard fashion. The puncture site over the left iliac crest was closed.

### Postoperative course

**Figure 4:**
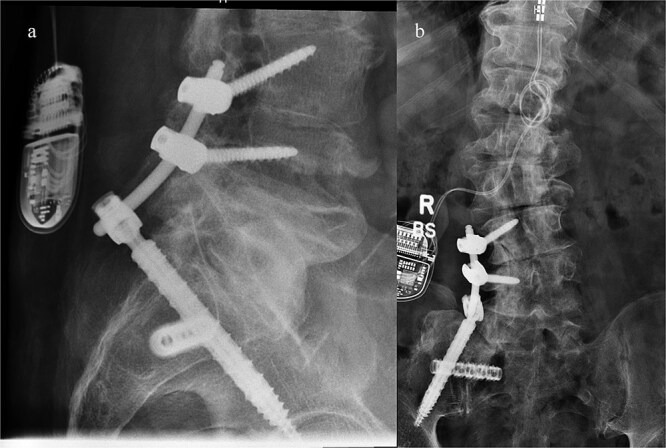
Postoperative lateral (left) and AP (right) re-demonstrated good placement of the hardware.

He was discharged home on postoperative day two, with instructions to remain toe-touch weight-bearing for three weeks. He was seen in the office two weeks after surgery. Lateral and antero-posterior pelvic radiographs showed good position of all hardware (Fig. 4). He reported mild incisional pain, but his preoperative pain had significantly improved. He was able to ambulate for the first time in years. Pain relief remains sustained three months after surgery.

## Discussion

SPECT-CT is a multimodal imaging technique that utilizes radioactive isotope tracers such as 99m-Technetium-Methylene diphosphonate (^99m^Tc-MDP), which is specifically used to examine bone abnormalities. The tracer accumulates at sites of injury, and uptake of ^99m^Tc-MDP is correlated with osteoblast differentiation [3]. Thus, increased activity may be seen in multiple settings, including malignancy, fracture, postoperative fusion site, or instability when the bone is attempting to fuse [4].

Pendleton and Ng showed a positive finding of facet arthropathy on SPECT-CT was associated with a more effective pain intervention compared to those diagnosed only with other radiologic modalities [2].

Varga *et al*. further studied the use of SPECT-CT through meta-analysis, concluding that a positive SPECT-CT finding of facet arthropathy was associated with a significantly higher facet blockade effect [5]. Due to paucity of formal studies, they were unable to draw adequate conclusions regarding the use of SPECT-CT and surgical outcomes.

The majority of the literature regarding use of SPECT-CT and spine pathology is related to postoperative pain [6, 7]. There are isolated case reports in which SPECT-CT was used to successfully identify the pain generator, similar to ours [4].

Russo *et al*. compared CT scans with SPECT-CT to evaluate the degree of structural facet joint degeneration in patients with low back pain [8]. While they found that 67% of patients with some degree of facet joint degeneration on CT demonstrated increased uptake in at least one facet joint on SPECT-CT, they found that >40% of the facet joint SPECT-CT uptake patterns did not degree with the correlation of degeneration on CT.

Spinal fusion for low back pain has largely fallen out of favor, with discogenic back pain often failing to improve long-term with fusion [9]. Spine surgeons may be tempted to discount patients as “non-surgical” in the absence of nerve compression, fracture, or frank instability on lumbar imaging [10]. However, this case demonstrates the role of further diagnostic work-up to identify not only a pain source but also a surgical treatment option.

Iliac screws have shown to create a more stable construct when used with lumbosacral pedicle screw fixation [11]. While this construct alone may have been sufficient in our case, the addition of SI joint fixation to improve stability has been corroborated in the literature [11]. The combination of lumbopelvic fixation combined with iliosacral screws, termed triangular osteosynthesis, is most commonly performed to treat sacral fractures [12]. Given the location of our patient’s particular pathology, the primary surgeon believed that this would be an appropriate surgical option.

## Conclusion

A creative approach to low back pain is crucial to properly manage patients with low back pain, especially those with debilitating pain that has failed to improve with extensive conservative measures. While no diagnostic tool is perfect, we encourage readers to employ the use of SPECT-CT as a diagnostic tool, particularly in cases when standard radiologic modalities do not clearly elucidate a pain-generating site. Osteoblastic lesions from degenerative changes may be unilaterally fused, with a goal of minimizing motion across the pain-generating segment, and can provide significant pain relief.

## References

[1] Hirsch BP, Sossamon J, Khan MA, et al. Applications of SPECT/CT in the evaluation of spinal pathology: a review. Int J Spine Surg 2024;18:9–23. 10.14444/8552 38050030 PMC11265499

[2] Pendleton J, Ng A. SPECT/CT scan: a new diagnostic tool in pain medicine. Curr Pain Headache Rep 2023;27:729–35. 10.1007/s11916-023-01177-4 37837482

[3] Zhong ZA, Peck A, Li S, et al. (99m)TC-methylene diphosphonate uptake at injury site correlates with osteoblast differentiation and mineralization during bone healing in mice. Bone Res 2015;3:15013. 10.1038/boneres.2015.13 26273540 PMC4472149

[4] Tender G, Constantinescu A, Conger A, et al. Primary pain generator identification by CT-SPECT in a patient with low back pain: a case report. BMC Res Notes 2017;10:132. 10.1186/s13104-017-2458-3 28327190 PMC5361848

[5] Varga M, Kantorová L, Langaufová A, et al. Role of single-photon emission computed tomography imaging in the diagnosis and treatment of chronic neck or back pain caused by spinal degeneration: a systematic review. World Neurosurg 2023;173:65–78. 10.1016/j.wneu.2023.02.058 36803686

[6] Al-Riyami K, Gnanasegaran G, Van den Wyngaert T, et al. Bone SPECT/CT in the postoperative spine: a focus on spinal fusion. Eur J Nucl Med Mol Imaging 2017;44:2094–104. 10.1007/s00259-017-3765-6 28681193

[7] Al-Riyami K, Vöö S, Gnanasegaran G, et al. The role of bone SPECT/CT in patients with persistent or recurrent lumbar pain following lumbar spine stabilization surgery. Eur J Nucl Med Mol Imaging 2019;46:989–98. 10.1007/s00259-018-4141-x 30191260

[8] Russo VM, Dhawan RT, Baudracco I, et al. Hybrid bone SPECT/CT imaging in evaluation of chronic low back pain: correlation with facet joint arthropathy. World Neurosurg 2017;107:732–8. 10.1016/j.wneu.2017.08.092 28847557

[9] Zhao L, Manchikanti L, Kaye AD, et al. Treatment of discogenic low back pain: current treatment strategies and future options-a literature review. Curr Pain Headache Rep 2019;23:86. 10.1007/s11916-019-0821-x 31707499

[10] Willems PC, Staal JB, Walenkamp GH, et al. Spinal fusion for chronic low back pain: systematic review on the accuracy of tests for patient selection. Spine J 2013;13:99–109. 10.1016/j.spinee.2012.10.001 23127364

[11] de Andrada PB, Lehrman JN, Sawa AGU, et al. Biomechanical effects of a novel posteriorly placed sacroiliac joint fusion device integrated with traditional lumbopelvic long-construct instrumentation. J Neurosurg Spine 2021;35:320–9. 10.3171/2020.11.SPINE201540 34144523

[12] Rovere G, De Mauro D, Smakaj A, et al. Triangular osteosynthesis and lumbopelvic fixation as a valid surgical treatment in posterior pelvic ring lesions: a systematic review. Front Surg 2024;11:1266393. 10.3389/fsurg.2024.1266393 38456170 PMC10917920

